# A Millimeter-Wave Dual-Band Substrate Integrated Waveguide Filter with Complementary Split Ring Resonators

**DOI:** 10.3390/mi17101105

**Published:** 2026-09-22

**Authors:** Yanling Wang, Xinrui Zhang, Guodong Su, Jincheng Wang, Jun Liu, Kailong Zhang

**Affiliations:** 1 School of Software, Northwestern Polytechnical University, Xi’an 710129, China; 2 Advanced Electronic Systems Laboratory, Beijing Institute of Aerospace Microsystem and Information Technology, Beijing 100094, China; 3 Microsystems R&D Center, Xi’an Microelectronics Technology Institute, Xi’an 710065, China; 4 Zhejiang Provincial Laboratory of Integrated Circuit Design, Hangzhou Dianzi University, Hangzhou 310018, China; guodong@hdu.edu.cn (G.S.);

**Keywords:** dual-band filter, substrate integrated waveguide (SIW), complementary split ring resonator (CSRR), millimeter waves

## Abstract

This paper presents a millimeter-wave dual-band bandpass filter based on a substrate integrated waveguide (SIW). The dual-band response is realized by embedding two pairs of complementary split-ring resonators (CSRRs) with different dimensions in the SIW cavity. Their geometry-dependent resonances introduce frequency-selective transmission zeros, reshaping the inherent high-pass response of the SIW into two passbands. The effects of the CSRR dimensions and loading sequence are investigated using several resonator configurations to clarify the formation and frequency control of the two bands. A prototype is fabricated using a wafer-level packaging process. The measured passbands are centered at 54.2 and 87.3 GHz, with insertion losses of 2.69 and 2.83 dB and fractional bandwidths of 21.8% and 13.3%, respectively. The measured results agree well with the simulations. The filter has a compact footprint of $\left(0.63\lambda_{g}\times 0.29\lambda_{g}\right)$.

## 1. Introduction

Filters are essential components of wireless communication systems, providing frequency-selective transmission and suppressing unwanted signals. As communication systems evolve toward higher frequencies, wider bandwidths, and greater integration, filters must combine low loss, high selectivity, sharp roll-off, strong out-of-band rejection, and compact size. Common implementations include planar coupled-resonator filters [1,2], conventional metallic waveguide filters, and substrate integrated waveguide (SIW) filters [3,4]. SIW structures provide waveguide-like field confinement through their top and bottom metal layers and via sidewalls. Compared with conventional planar transmission lines such as microstrip and coplanar waveguide, SIW generally offers a higher quality factor, lower radiation loss, and greater power-handling capability, while retaining a planar and readily integrated configuration. Although its performance is typically inferior to that of hollow metallic waveguides because of dielectric and conductor losses, this balance makes SIW attractive for millimeter-wave filter design.

Extensive studies have been conducted on SIW bandpass filter structures, and various high-performance implementations have been proposed [5,6,7]. However, higher-order and parasitic modes often produce spurious passbands near the main passband, complicating their suppression and limiting the use of SIW filters in wideband, high-rejection applications [8].

Existing single-passband SIW filters have mainly focused on improving bandwidth, selectivity, stopband suppression, and circuit size. Band-stop loading elements have been introduced to generate additional transmission zeros and suppress spurious responses [9]. In [10], U-shaped slots were etched in SIW cavities to excite multiple resonant modes, resulting in a compact fifth-order wideband bandpass filter. In [11], capacitive quasi-coplanar waveguide feeding structures and complementary split-ring resonators (CSRRs) loading were employed to excite an evanescent-mode SIW response below the cutoff frequency, achieving a compact Q-band filter with a wide passband and low insertion loss.

For dual- and multiband operation, several different mechanisms have been investigated. The inherent spurious passband of an SIW cavity can be utilized as a second passband [12], while dual-band responses can also be generated by coupling degenerate cavity modes, such as the *TE*_102_ and *TE*_201_ modes [13]. Beyond their use for single-passband miniaturization in [11], CSRRs can serve as additional resonant elements in SIW cavities. Because their resonant frequencies are primarily determined by their geometrical dimensions, multiple CSRRs can be used to generate distinct passbands and provide flexible frequency control [14,15].

Miniaturized SIW configurations have also been investigated. For example, a half-mode SIW structure was employed in [16] to reduce the circuit footprint, although this reduction was accompanied by increased insertion loss and a lower quality factor. Therefore, realizing a compact dual-band SIW filter while maintaining low loss, high selectivity, independently controllable passbands, and satisfactory out-of-band rejection remains a significant design challenge.

Moreover, the development of chiplet-based heterogeneous integration and 3D packaging has increased the demand for RF passive components that can be directly implemented using wafer-level fabrication processes [11,17]. In addition to electrical performance, such components must satisfy the requirements of process manufacturability, integration density, and electromagnetic isolation in 3D heterogeneous systems. Achieving low loss and stable high-frequency performance within a limited footprint remains a key challenge.

Leveraging the fabrication capabilities of wafer-level packaging (WLP) technology and the band-stop resonance of CSRRs, this work proposes a millimeter-wave dual-band SIW filter loaded with two pairs of differently sized CSRRs. The CSRRs are symmetrically embedded in the SIW cavity with a fixed spacing, introducing two geometry-dependent transmission zeros into the inherent high-pass response of the SIW and thereby forming two distinct passbands. Because the resonant frequency of each CSRR pair is primarily determined by its geometric dimensions, the two transmission zeros and the resulting passbands can be flexibly controlled by independently adjusting the corresponding CSRR sizes. The fixed-spacing configuration also helps reduce mutual coupling between the differently sized resonators, improving the stability and controllability of the dual-band response. To evaluate the proposed design strategy, a prototype is fabricated using a WLP process and experimentally characterized. The following sections systematically investigate the operating principle, independent control and mutual coupling of the CSRRs, higher-order CSRR configuration, loss mechanisms, and comparison between the simulated and measured responses.

The remainder of this paper is organized as follows. Section 2 focuses on the analysis of the design principles and implementation methods of the SIW dual-band filter loaded with CSRRs of different sizes. Section 3 presents the simulation and measurement results, along with a comparative analysis of the key performance indicators. Finally, the work is summarized, and future optimization directions are discussed.

## 2. Analysis and Design

### 2.1. SIW with Asymmetrically Arranged CSRRs

An SIW emulates a dielectric-filled rectangular waveguide and inherently exhibits a high-pass response. For the dominant *TE*_10_ mode, waves propagate only above the cutoff frequency; below cutoff, the fields decay exponentially and cannot effectively transmit power. The cutoff frequency and *TE*_10_-mode transmission-pole frequencies are expressed as follows [18,19]:

(1) $$\begin{array}{c} f_{cutoff-{TE}_{10}}=\frac{c}{2\sqrt{{\mu_{r}\varepsilon }_{r}}}{\left(W-\frac{D^{2}}{0.95S}\right)}^{-1} \end{array}$$

(2) $$\begin{array}{c} f_{TP-{TE}_{10}}=\frac{c}{2\sqrt{\mu_{r}\varepsilon_{r}}}\sqrt{{\left(W-\frac{D^{2}}{0.95S}\right)}^{-2}+n^{2}{\left(L-\frac{D^{2}}{0.95S}\right)}^{-2}} \end{array}$$

where *c* is the speed of light in vacuum, *n* denotes the longitudinal mode index, *W* and *L* are the width and length of the SIW cavity, respectively, *D* is the via diameter, and *S* is the center-to-center spacing between adjacent vias. Based on the theoretical calculations and subsequent full-wave simulation refinement, the final parameters were selected as D=50 μm, S=165 μm, L=2000 μm, and W=1450 μm. The full-wave electromagnetic simulations were performed using CST Studio Suite. The dielectric substrate was modeled using the same parameters as those employed in fabrication: a relative permittivity of 3.2, a loss tangent of 0.009, and a thickness of 14.6 μm. Figure 1 illustrates the basic SIW structure and its transmission characteristics.

Following the determination of the basic SIW dimensions, two CSRRs with different geometrical dimensions are introduced into the SIW cavity, as shown in Figure 2a. The two resonators, denoted as CSRR1 and CSRR2, are etched on the top metal layer of the SIW and arranged asymmetrically with respect to the cavity center. Their detailed geometrical parameters are illustrated in Figure 2b. Owing to the geometry-dependent band-stop resonance of the CSRR, the two resonators generate transmission zeros at different frequencies. In particular, the larger CSRR1 exhibits a lower resonant frequency, whereas the smaller CSRR2 resonates at a higher frequency. This configuration therefore provides the basis for introducing two separately controlled stopband resonances into the inherent high-pass response of the SIW.

To clarify the contribution of each resonator to the overall frequency response, three loading configurations are simulated under otherwise identical conditions: the SIW loaded only with CSRR1, the SIW loaded only with CSRR2, and the SIW loaded with both resonators. The corresponding simulated $S_{21}$ responses are compared in Figure 3. When only CSRR1 is loaded, a lower-frequency transmission zero, denoted as TZ1, is generated. By contrast, loading only CSRR2 produces a higher-frequency transmission zero, denoted as TZ2. When both resonators are simultaneously loaded, the two transmission zeros are retained in the response, with only limited frequency deviations compared with the individually loaded cases.

The comparison demonstrates that TZ1 is predominantly controlled by CSRR1, whereas TZ2 is mainly determined by CSRR2. The limited displacement of the transmission zeros after simultaneous loading also indicates relatively weak interaction between the two resonators. Consequently, the two CSRR-induced stopband resonances reshape the inherent high-pass response of the SIW, suppressing transmission at the corresponding frequencies and forming two distinct passbands. Therefore, the dual-band response is achieved through the combined effect of the SIW high-pass characteristic and the geometry-dependent resonances of the two differently sized CSRRs.

### 2.2. Weak Coupling and Quasi-Independent Frequency Control

Because CSRR1 and CSRR2 are simultaneously loaded in the same SIW cavity, electromagnetic coupling between them may perturb their resonant frequencies [15]. To evaluate the interaction between the two resonators and determine whether their transmission zeros can be controlled separately, two cross-parametric simulations were performed. Throughout the analysis, the center positions of the resonators and their center-to-center spacing *f* = 500 μm were kept unchanged. First, $r_{2}$ was fixed at 160 μm while $r_{1}$ was varied from 200 to 250 μm. Subsequently, $r_{1}$ was fixed at 200 μm while $r_{2}$ was varied from 160 to 180 μm. All other structural and material parameters remained unchanged. Therefore, the shift in the transmission zero associated with the varied CSRR represents its direct frequency sensitivity, whereas the shift in the other transmission zero reflects the cross-coupling between the two resonators.

Figure 4a presents the simulated responses obtained by varying $r_{1}$. The corresponding transmission-zero frequencies are summarized in Table 1. As $r_{1}$ increases from 200 to 250 μm, TZ1 shifts monotonically from 72.9 to 47.1 GHz, corresponding to a total frequency variation of 25.8 GHz. This downward shift is consistent with the CSRR resonance mechanism, because increasing the resonator dimensions increases its effective inductance and capacitance and consequently reduces its resonant frequency. Over the same range, TZ2 changes only from 111.5 to 113.7 GHz, with a total variation of 2.2 GHz. The average direct sensitivity of TZ1 to $r_{1}$ is approximately −0.516 GHz/μm, whereas the cross-sensitivity of TZ2 is only 0.044 GHz/μm, corresponding to approximately 8.5% of the direct sensitivity. These results indicate that $r_{1}$ predominantly controls TZ1 and has only a limited influence on TZ2.

The cross-parametric simulations obtained by varying the radius of CSRR2 while keeping CSRR1 unchanged is presented in Figure 4b, and the corresponding transmission-zero frequencies are summarized in Table 2. When $r_{2}$ increases from 160 to 180 μm, TZ2 shifts from 111.8 to 90.2 GHz, resulting in a total variation of 21.6 GHz. In contrast, TZ1 remains between 72.9 and 73.5 GHz, with a maximum variation of only 0.6 GHz. The maximum cross-variation in TZ1 is therefore less than 2.8% of the direct frequency variation in TZ2. This result confirms that $r_{2}$ mainly determines TZ2, while its influence on TZ1 is comparatively weak.

The two cross-parametric simulations provide quantitative evidence that the two resonators exhibit weak electromagnetic interaction at the selected spacing. Although the small residual shifts in the unaffected transmission zeros indicate that the resonators are not completely decoupled, the coupling is sufficiently weak to permit quasi-independent adjustment of TZ1 and TZ2. Accordingly, the final values are selected as $r_{1}$ = 200 μm, $r_{2}$ = 160 μm, and *f* = 500 μm. This configuration produces two well-separated transmission zeros near 73 and 112 GHz, which reshape the inherent high-pass response of the SIW and provide the basis for realizing the desired dual-passband response.

### 2.3. SIW with Higher-Order CSRRs

Although the first-order CSRR loading generates the required transmission zeros, its resonant strength is insufficient to provide the desired stopband rejection. To enhance the suppression performance without substantially changing the transmission-zero positions determined in Section 2.2, a second-order CSRR configuration is introduced. As shown in Figure 5a, each CSRR type is extended from a single resonator to a pair of identical resonators. The two resonators in each pair are arranged symmetrically about the center of the SIW cavity, and their center-to-center spacing is denoted by q. The previously determined radii $r_{1}$ and $r_{2}$, as well as the relative positions of the two differently sized resonant groups, remain unchanged.

Figure 5b compares the simulated $S_{21}$ responses of the first- and second-order CSRR configurations. Both structures retain the two passbands and generate transmission zeros at approximately the same frequency positions. However, the second-order configuration increases the rejection level of both transmission zeros to approximately −40 dB, compared with about −28 and −34 dB for the first-order configuration. Meanwhile, the transmission characteristics within the two passbands remain largely unchanged. These results demonstrate that the additional CSRRs enhance the equivalent resonant strength and improve the stopband rejection without significantly disturbing the intended dual-passband response.

The spacing q determines the electromagnetic interaction within each second-order CSRR pair and therefore affects both the resonant suppression and passband matching. To determine an appropriate value, q was varied from 500 to 700 μm while all other structural parameters were kept unchanged. The simulated $S_{21}$ responses are compared in Figure 6a. As q increases, the two transmission-zero frequencies remain relatively stable, whereas their rejection levels increase noticeably. At q = 500 μm, the two transmission zeros exhibit rejection levels of approximately −34 and −32 dB. Increasing q to 700 μm deepens them to approximately −44 and −45 dB. This observation indicates that q mainly controls the resonant strength of the paired CSRRs, while the transmission-zero frequencies are still predominantly determined by the CSRR dimensions.

However, the $S_{11}$ responses in Figure 6b show that maximizing the transmission-zero depth does not necessarily provide the best overall filter performance. Although q = 700 μm produces the strongest stopband suppression, the impedance matching in the lower passband deteriorates, particularly around 57–63 GHz, where $S_{11}$ approaches or exceeds −10 dB. In contrast, q = 500 μm provides weaker rejection at both transmission zeros. When q = 600 μm, the transmission-zero rejection levels remain approximately −39 and −37 dB, while relatively balanced impedance matching is maintained in both passbands. Therefore, q = 600 μm is selected as a suitable compromise between passband matching and stopband rejection.

Finally, stepped microstrip-to-SIW transitions are introduced at both ports, as illustrated in Figure 5a, to facilitate mode conversion between the quasi-TEM mode of the microstrip line and the dominant TE mode of the SIW. Ground–signal–ground (GSG) probe pads are also incorporated to support subsequent on-wafer measurements. The optimized structural parameters of the filter are listed in Table 3.

### 2.4. Loss Consideration

Since the CSRR slots etched on the top metal layer may introduce electromagnetic leakage, the loss characteristics of the proposed filter are further investigated. Following the normalized loss evaluation method reported in [9], three simulation configurations are considered while maintaining identical geometrical dimensions, boundary conditions, ports, and mesh settings. In the radiation-only case, the conductors are modeled as perfect electric conductors (PECs), and the dielectric loss tangent is set to zero. The dielectric-and-radiation-loss case uses PECs together with the actual dielectric loss tangent of 0.009, whereas the total-loss case includes radiation, dielectric, and conductor losses. The normalized power loss is calculated from the simulated S-parameters as

(3) $$\begin{array}{c} L_{\mathrm{norm}}=1-S_{11}S_{11}^{*}-S_{21}S_{21}^{*} \end{array}$$

Figure 7 compares the calculated normalized power losses under three cumulative loss configurations: radiation loss only, radiation plus dielectric loss, and total loss including radiation, dielectric, and conductor losses. The radiation-only loss remains below 0.063 and 0.043 within the first and second passbands, respectively. At the two center frequencies of 54.2 and 87.3 GHz, the normalized radiation-only losses are approximately 0.050 and 0.029, respectively. When dielectric loss is added, the cumulative normalized losses increase to approximately 0.305 and 0.360. After conductor loss is further included, the total normalized losses reach 0.395 and 0.492, respectively. Therefore, the differences between the cumulative values correspond to dielectric-loss contributions of approximately 0.255 and 0.331 and conductor-loss contributions of approximately 0.090 and 0.132 at the two center frequencies. These results indicate that radiation leakage through the CSRR slots is limited, dielectric dissipation is the dominant loss mechanism, and conductor loss makes a smaller but non-negligible contribution.

## 3. Measurement Results

To experimentally validate the proposed method, the dual-band SIW bandpass filter was fabricated using wafer-level packaging technology. A photograph of the fabricated filter is shown in Figure 8. Its core size is 1600 μm × 2000 μm, demonstrating a compact implementation. S-parameter measurements were performed using a Keysight N5290A vector network analyzer (Keysight Technologies, Santa Rosa, CA, USA) and a Cascade Microtech probe station (Cascade Microtech, Beaverton, OR, USA; now FormFactor, Livermore, CA, USA) equipped with GSG probes. On-wafer calibration was performed using a standard short-open-load-thru (SOLT) technique on an impedance standard substrate (ISS) to establish the reference plane at the probe tips. The simulated and measured S-parameter responses of the proposed filter are compared in Figure 9.

The results indicate that the center frequencies are approximately 54.2 GHz and 87.3 GHz, respectively, and the return loss in both simulation and measurement is better than 10.1 dB within the passband. At the first center frequency, the simulated insertion loss is 2.44 dB, while the measured value is 2.69 dB. At the second center frequency, the simulated insertion loss is 2.62 dB, while the measured value is 2.83 dB. Both simulation and measurement show that the two transmission zeros generated by CSRRs of different dimensions are located near 70 GHz and 108 GHz, respectively. The simulated passbands are 48.0–59.9 GHz and 82.1–93.8 GHz, while the measured passbands are 48.3–60.1 GHz and 82.5–94.1 GHz. The slight frequency deviation between simulation and measurement is mainly attributed to the fabrication tolerance of wafer-level packaging (WLP) technology in the millimeter-wave band and the sensitivity of structural dimensions to processing inaccuracies. Overall, the measured results confirm the feasibility and stability of the proposed design methodology.

Table 4 compares the proposed filter with recently reported dual-band filters. Compared with [12], the proposed design provides wider operating bandwidths, while it achieves a smaller normalized circuit size than [15]. These results demonstrate a competitive trade-off among bandwidth, compactness, and dual-band performance.

## 4. Conclusions

This paper presents a compact millimeter-wave dual-band SIW bandpass filter loaded with differently sized CSRRs. The fabricated filter operates at center frequencies of 54.2 and 87.3 GHz, with measured 3 dB passbands of 48.3–60.1 GHz and 82.5–94.1 GHz and corresponding insertion losses of 2.69 and 2.83 dB, respectively. Independent adjustment of the CSRR dimensions enables control of the two transmission zeros, while the higher-order CSRR configuration enhances stopband rejection. The compact footprint of 0.63 * 0.29 λ_g_^2^, together with the controlled and relatively low insertion loss at millimeter-wave frequencies, demonstrates the practical potential of the proposed filter for compact integrated dual-band systems.

## Figures and Tables

**Figure 1:**
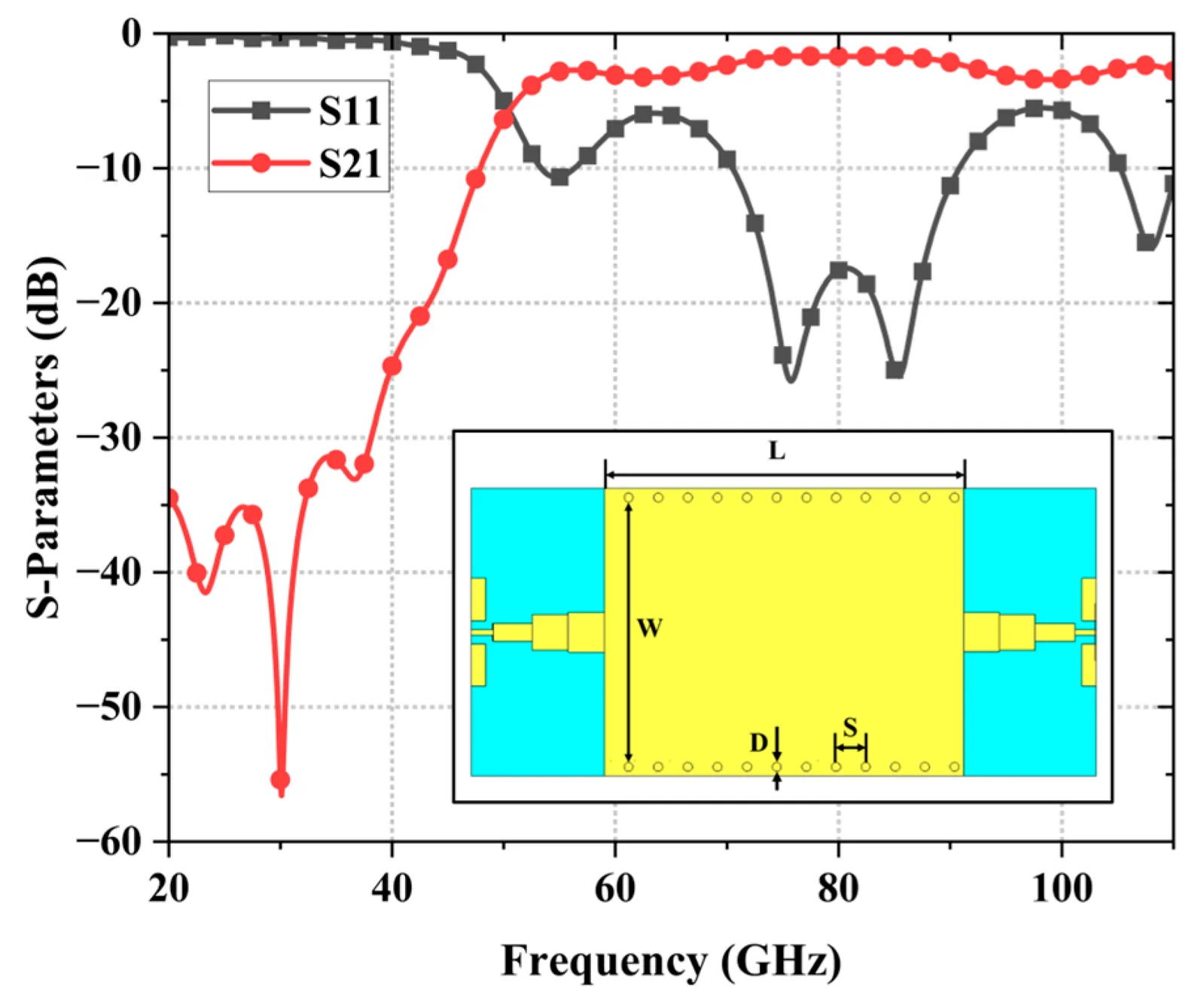
The structure and the simulated S-parameters of the SIW.

**Figure 2:**
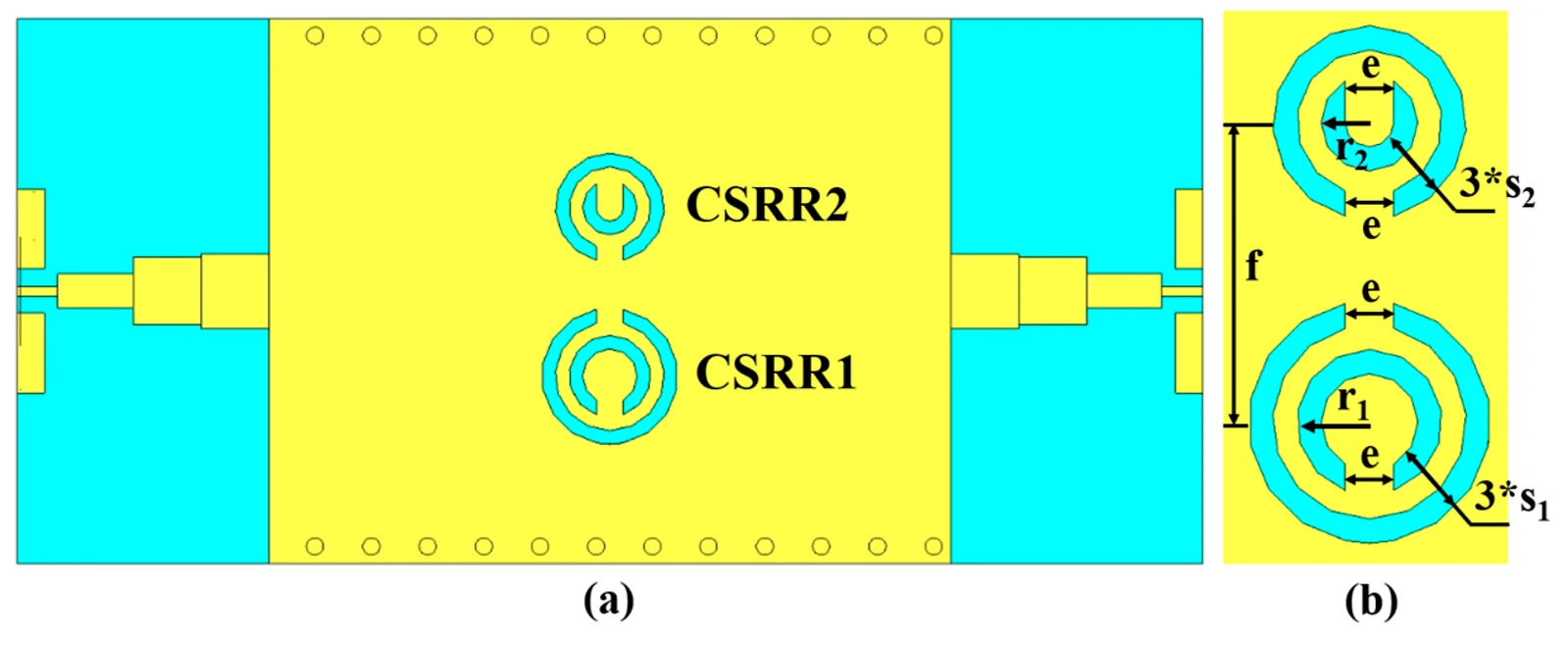
(**a**) SIW loaded with a pair of asymmetrically arranged CSRRs; (**b**) Dimensional parameters of the CSRR.

**Figure 3:**
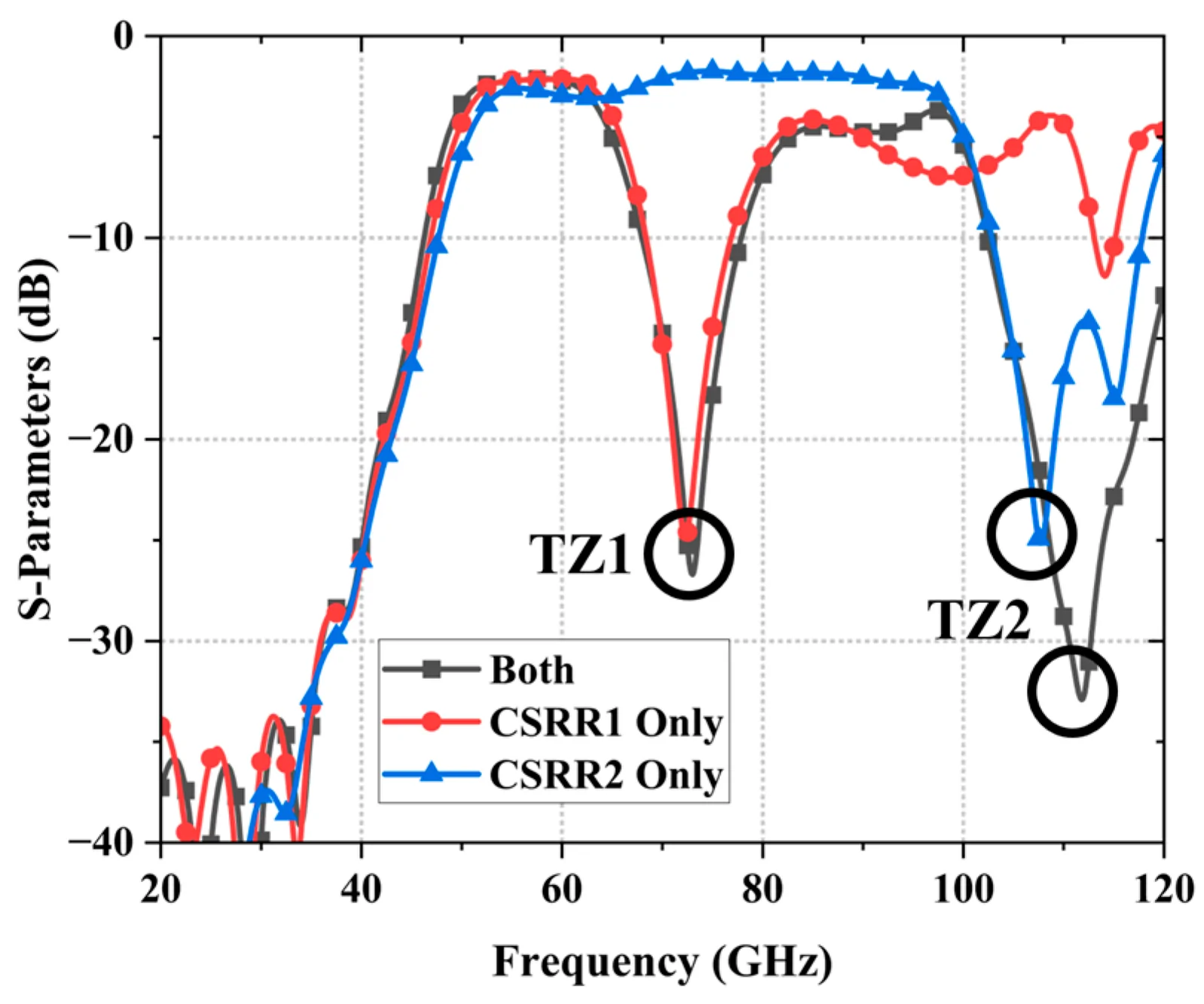
Simulated $S_{21}$ responses of the SIW with different CSRR loading configurations.

**Figure 4:**
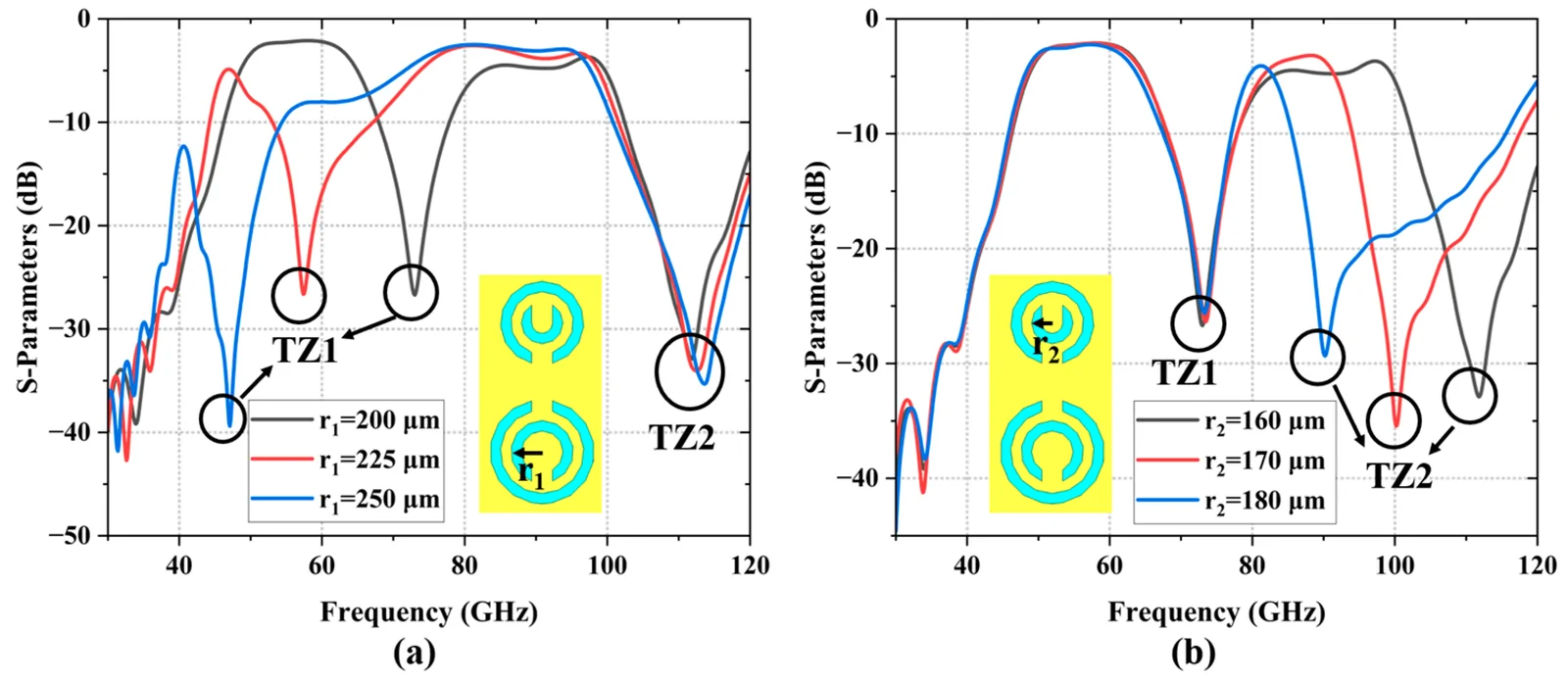
(**a**) Effect of the CSRR1 radius ($r_{1}$) on the simulated transmission response with $r_{2}=160$ μm and f=500 μm. (**b**) Effect of the CSRR2 radius ($r_{2}$) on the simulated transmission response with $r_{1}=200$ μm and f=500 μm.

**Figure 5:**
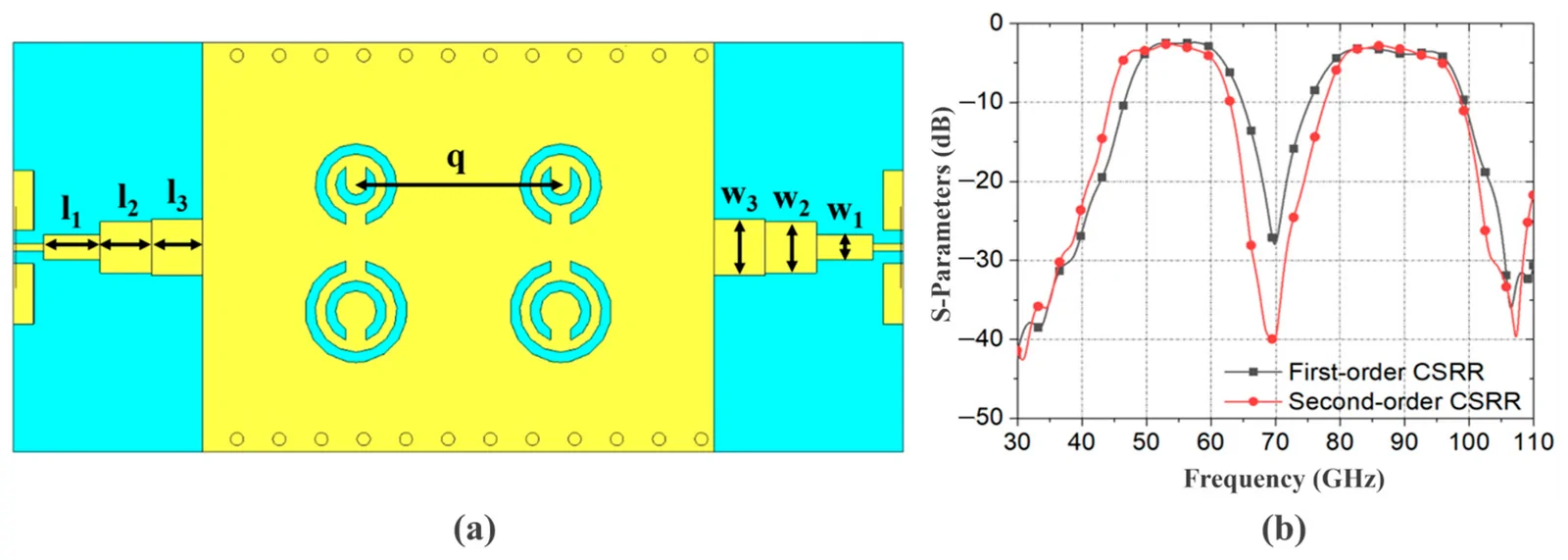
Designed CSRR structure and simulation results. (**a**) Designed second-order CSRR structure; (**b**) Comparison of simulation results between first-order CSRR and second-order CSRR.

**Figure 6:**
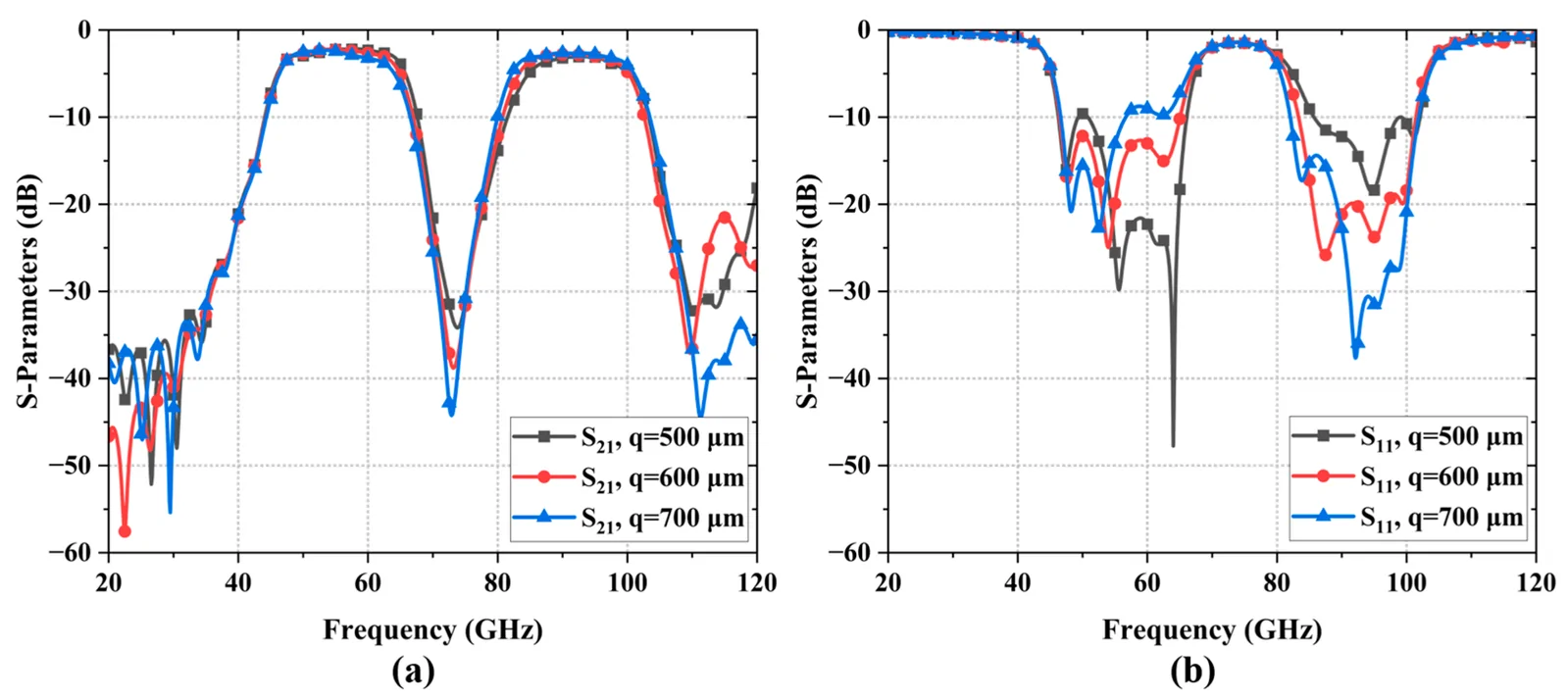
Effect of the center-to-center spacing q between the paired CSRRs on the simulated S-parameters of the second-order CSRR-loaded SIW filter: (**a**) $S_{21}$; (**b**) $S_{11}$.

**Figure 7:**
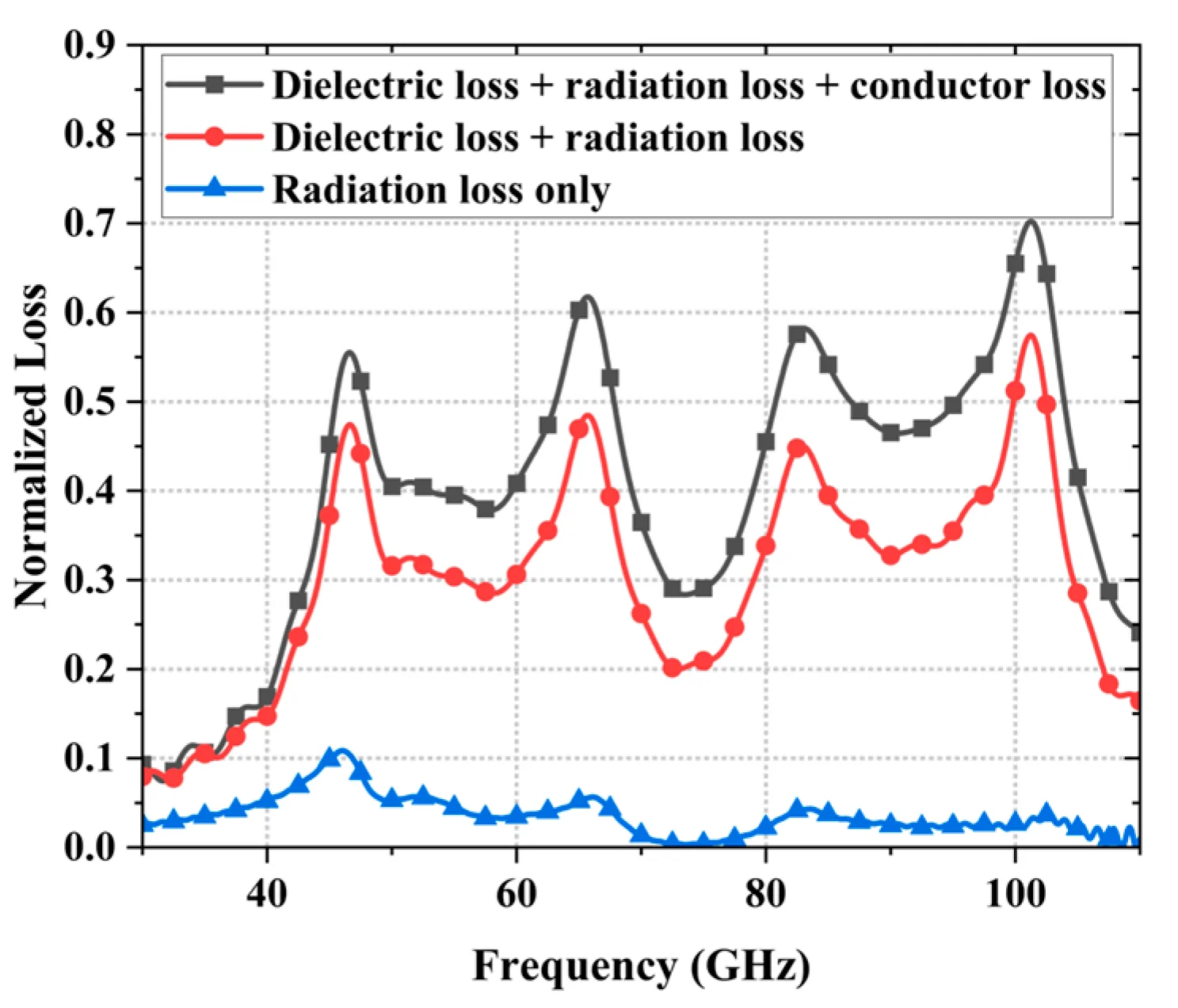
Calculated normalized losses of the proposed CSRR-loaded SIW filter under different loss configurations.

**Figure 8:**
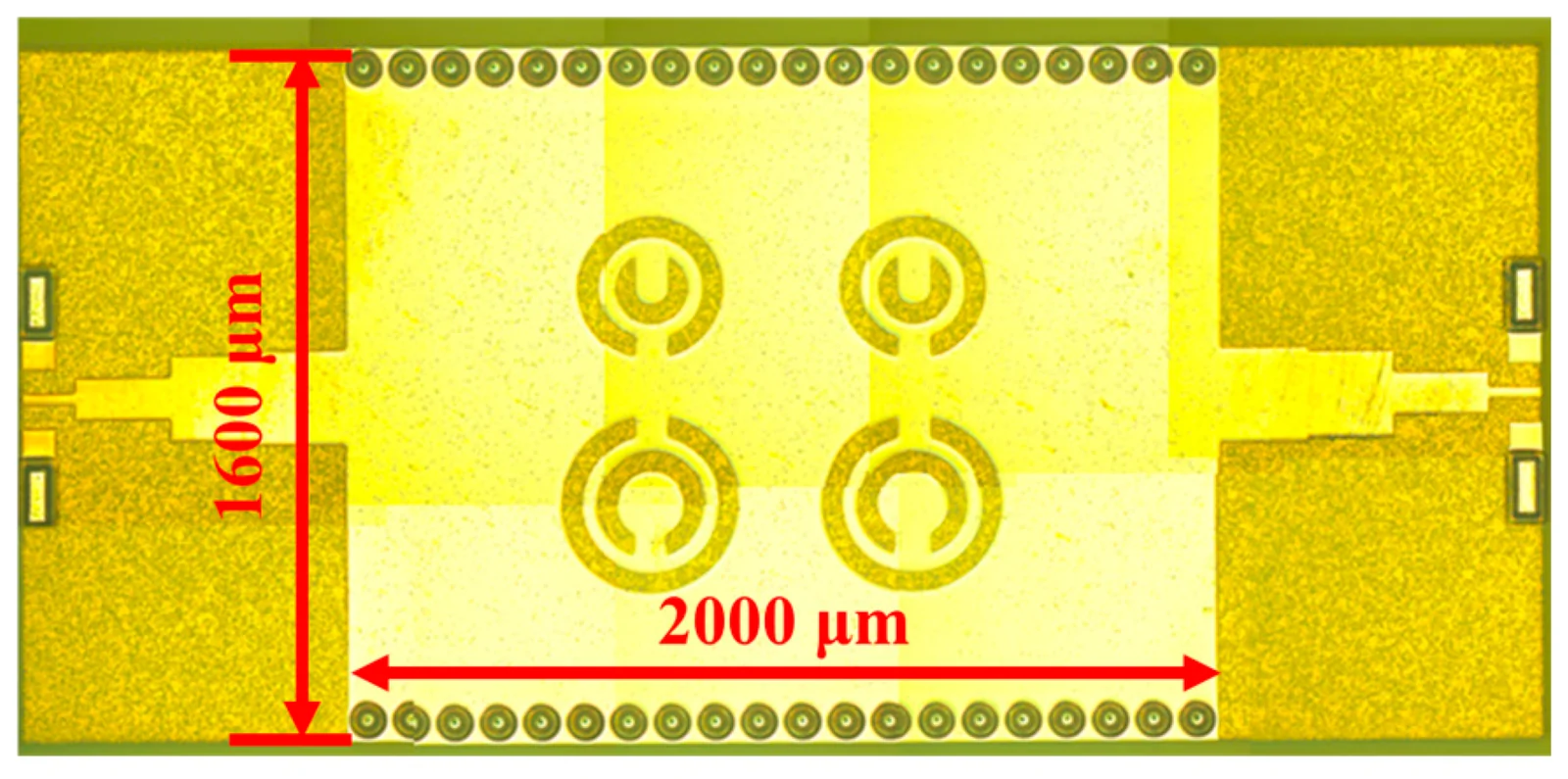
Fabricated CSRR filters.

**Figure 9:**
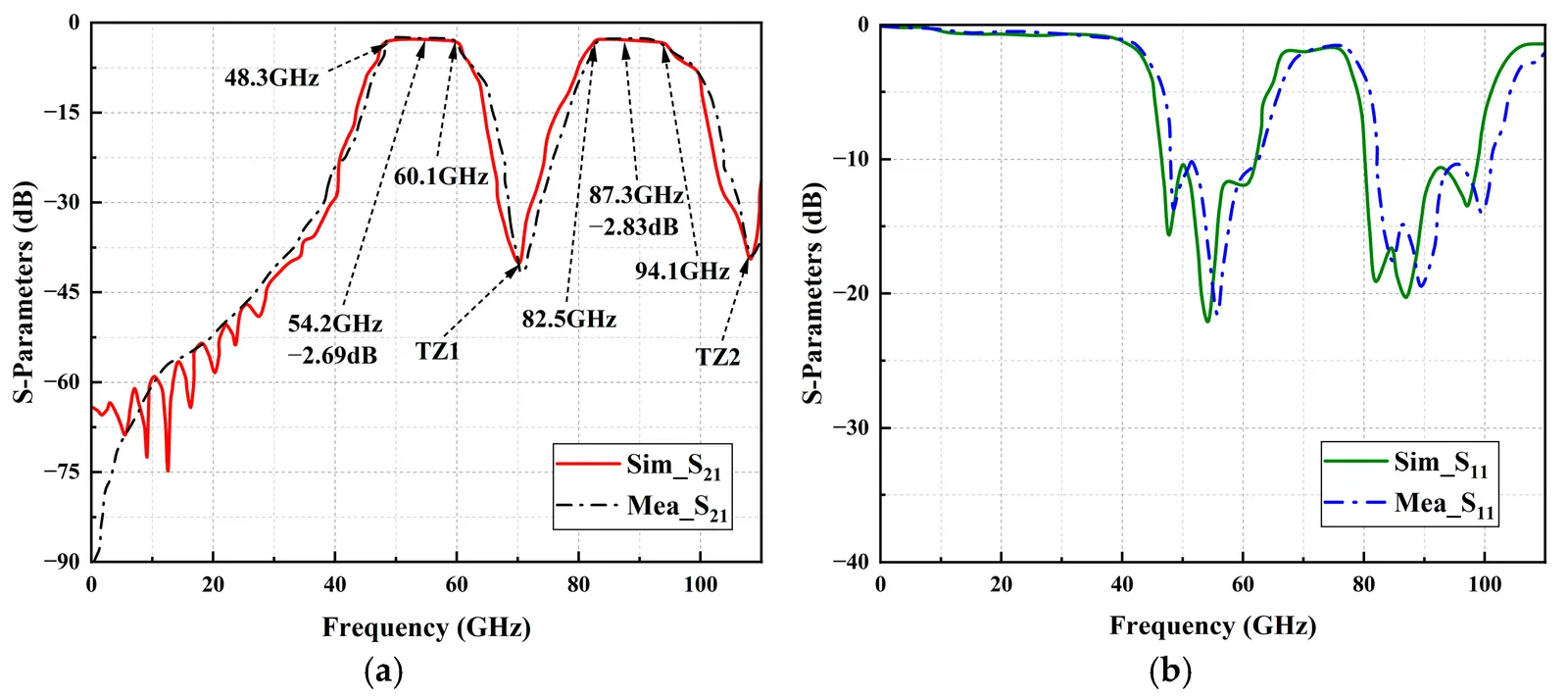
Comparison between measured and simulated results. (**a**) $S_{21}$; (**b**) $S_{11}$.

**Table 1:** Simulated TZ frequencies for different radii of CSRR1 ($r_{2}=160$ μm and f=500 μm).

Radius of the CSRR1 (μm)	TZ1	TZ2
200	72.9 GHz	111.5 GHz
225	57.4 GHz	112.2 GHz
250	47.1 GHz	113.7 GHz

**Table 2:** Simulated TZ frequencies for different radii of CSRR2 ($r_{1}=200$ μm and f=500 μm).

Radius of the CSRR2 (μm)	TZ1	TZ2
160	72.9 GHz	111.5 GHz
170	73.5 GHz	100.2 GHz
180	73.1 GHz	90.2 GHz

**Table 3:** Structural parameters of the proposed SIW dual-band filter. (Units: μm).

Parameters	Values	Parameters	Values
W1	100	L1	220
W2	200	L2	200
W3	220	L3	200
*r*_1_	200	s1	40
*r*_2_	160	s2	40
*f*	500	q	600
e	80		

**Table 4:** Performance comparison of filters.

Ref.	Frequency (GHz)		FBW (%)		IL (dB)		Size (λ_g_ × λ_g_)
	*f*_1_	*f*_2_	BW1	BW2	*f*_1_	*f*_2_	
[12]	8	10	3.5	6.0	2.25	1.92	0.65 × 0.65
[15]	6.22	8.24	7.7	3.9	0.86	1.32	0.89 × 0.41
[20]	3.6	6.4	3.3	2.4	1.3	1.8	0.29 × 0.58
[21]	28.3	38.5	2.4	3.4	≤1.0	≤1.0	0.82 × 0.45
[22]	26.75	31.55	3.0	1.6	2.64	4.20	1.34 × 2.55
TW.	54.2	87.3	21.8	13.3	2.69	2.83	0.63 × 0.29

## Data Availability

The original contributions presented in this study are included in the article. Further inquiries can be directed to the corresponding author.

## Abbreviations

The following abbreviations are used in this manuscript:

SIW	Substrate integrated waveguide
CSRR	Complementary split ring resonators
WLP	Wafer-level packaging
TZ	Transmission zero
TEM	Transverse electromagnetic
TE	Transverse electric
FBW	Fractional bandwidth
IL	Insertion loss
